# Editorial: Functional fitness/high intensity functional training for health and performance

**DOI:** 10.3389/fphys.2022.1024809

**Published:** 2022-09-14

**Authors:** M. A. Soriano, D. Boullosa, F. Amaro-Gahete

**Affiliations:** ^1^ Strength Training and Neuromuscular Performance Research Group (STreNgthP), Camilo José Cela University, Madrid, Spain; ^2^ Federal University of Mato Grosso do Sul, Campo Grande, Brazil; ^3^ Collegue of Healthcare Sciences, James Cook University, Townsville, QLD, Australia; ^4^ Research and Development Department, iLOAD Solutions, Campo Grande, Brazil; ^5^ EFFECTS Research Group, Department of Physiology, Faculty of Medicine, University of Granada, Granada, Spain; ^6^ PROFITH “PROmoting FITness and Health Through Physical Activity” Research Group, Department of Physical Education and Sport, Faculty of Sport Sciences, University of Granada, Granada, Spain

**Keywords:** CrossFit ®, high-intensity interval training, resistance exercise, multimodal training, cross-training

## Introduction

Functional fitness training (FFT) is an emerging fitness trend that emphasizes functional, multi-joint movements, including aerobic (e.g., cycling, rowing, running) and strength exercises (e.g., weightlifting and derivatives: squat, snatch, clean and jerk, bench press, deadlift; bodyweight exercises: air squat, push-up, pull-up, muscle-up; plyometrics: box jumps, tuck ups) (Claudino et al., 2018; Feito et al., 2018). Researchers have shown that FFT may be not only suitable for professional athletes but also for populations with different fitness levels. Indeed, it is suggested that FFT elicits greater muscle recruitment than aerobic exercises alone, thereby improving both endurance and muscular strength and power (Bergeron et al., 2011; Claudino et al., 2018; Feito et al., 2018; Schlegel, 2020; Sharp et al., 2022). However, FFT units (i.e., workouts) are highly varied daily, and more research is needed to clarify its acute effects and its associated chronic training adaptations (Bergeron et al., 2011; Claudino et al., 2018; Feito et al., 2018; Schlegel, 2020; Sharp et al., 2022). Therefore, the aim of this Research Topic is to increase the knowledge of the evidence-based effects and adaptations of implementing FFT on health and performance in individuals with different biological conditions.

## Terminology

CrossFit^®^ has been used in research and practice to denominate FFT as a fitness trend. Importantly, the CrossFit^®^ company (CrossFit^®^ Inc., LLC) has revolutionized the fitness industry achieving, to date, more than 11,000 affiliated boxes worldwide and implemented competitiveness with the inclusion of structured competitions such as the “*CrossFit Games”* (Kuhn, 2013; Claudino et al., 2018; Dexheimer et al., 2019; Glassman, 2020; Martínez-Gómez et al., 2020). Furthermore, the CrossFit^®^ brand is worth $4 billion, according to the prestigious Forbes journal (Ozanian, 2015). However, since CrossFit^®^ is a registered brand and not an actual exercise modality, several terms have also been used in the scientific literature, along with CrossFit^®^, to equally denominate this fitness trend: high-intensity multimodal training, extreme conditioning programs, functional fitness training, high-intensity functional training, and mixed modal training (Bergeron et al., 2011; Feito et al., 2018; Marchini et al., 2019; Sharp et al., 2022).

An opinion article attempted to solve the problem with the terminology found in the scientific literature since the inclusion of FFT in the fitness industry worldwide. According to Dominski et al., FFT is the most comprehensive and inclusive term. It should be adopted both in research and practice since it is based on functional training and physical fitness terms to describe various characteristics and activities performed. Then, FFT is characterized by a variety of movement patterns (e.g., knee or hip dominant exercises, pull, push), activities (e.g., weightlifting, strength, gymnastics, metabolic and aerobic conditioning), and energy systems used (e.g., ATP-CP/phosphagen, glycolytic, and oxidative) (Dominski et al.). Subsequently, FFT should develop participants’ competencies in aerobic capacity, strength, bodyweight endurance and skills, and power development (Dominski et al.).

## Acute effects of functional fitness training

FFT is characterized by a wide variety of workouts, which differ in training duration (i.e., volume) and intensity (Claudino et al., 2018; Feito et al., 2018; Tibana and Frade De Sousa, 2018; Schlegel, 2020; Sharp et al., 2022). Training preparation and performance of FFT are usually connected with the principles of concurrent training, usually combining endurance-oriented (e.g., cycling, rowing, running) and strength-oriented (e.g., weightlifting, bodyweight exercises) activities within the workouts (Schlegel, 2020). Researchers have reported that FFT sessions induce remarkable fatigue levels with impairments in performance indicators and elevated levels of perceived effort (Tibana and Frade De Sousa, 2018; Schlegel, 2020; Dominski et al.). Furthermore, it is frequently reported that participants show high metabolic and cardiovascular stress (e.g., elevated blood lactate concentration and heart rate), high rates of perceived exertion, and elevated immune and hormonal responses (e.g., testosterone, cortisol, IL-6, IL-10) to FFT workouts (Tibana and Frade De Sousa, 2018; Schlegel, 2020; Sharp et al., 2022). Nonetheless, more research is needed to increase the knowledge of the acute effects of implementing FFT on health and performance.

In this Research Topic, two articles focused on the acute effects of FFT with important practical applications (Machado et al.; Saeterbakken et al.). Saeterbakken et al. demonstrated that performing the bench press throw (BPT) exercise using a bouncing technique (i.e., allowing the barbell to rebound off the chest) increased average power output (7.9–14.1%, *p* ≤ 0.001, ES = 0.48–0.90), average velocity (6.5–12.1%, *p* ≤ 0.001, ES = 0.48–0.91), and decreased time to peak power (11.9–31.3%, *p* ≤ 0.001–0.05, ES = 0.33–0.83) across a battery of loads (30–60 kg) in 27 resistance-trained men. In addition, descending the barbell with a higher velocity increased the power outputs, velocity, and time to peak power (*p* ≤ 0.001–0.003) during the subsequent BPT ascending phase. In theory, the athletes could use these findings to increase effectiveness and improve performance during workouts and benchmarks that implement the bench press exercise (e.g., “LINDA”).

Machado et al. demonstrated that exercise distribution using body weight high-intensity interval training (HIIT)-based workouts promote alterations in training load parameters. In this study, 20 male participants performed three 20-min workouts, consisting of 20 sets of 30 s of body weight complexes performed at maximal intensity, followed by 30 of passive recovery. Three training designs matched the exercises but differed in order: A) jumping jack, burpee, mountain climb, and squat jump; B) jumping jack, mountain climb, burpee, and squat jump; C) burpee, squat jump, jumping jack, and mountain climb. The main findings of this study were that participants of design A performed significantly more repetitions (26–36%, *p* < 0.001) and had higher values for perceived recovery (19–73%, *p* < 0.001) despite no significant differences were found between protocols for relative heart rate, perceived exertion, and lactate concentration (*p* > 0.05). Therefore, based upon these results, participants of FFT are encouraged to adapt the exercise order during bodyweight HIIT-based workouts to improve their performance and recovery perceptions.

Neto et al. and Martínez-Gómez et al. aimed to study the time course of recovery and different recovery strategies after FFT, respectively. On the one hand, Neto et al. described the acute and delayed time course of recovery of eight trained male participants following the CrossFit^®^ benchmark “KAREN” (i.e., 150 wall balls for time using a 9 kg med ball, aiming to hit a target 3 m high). Creatine kinase (CK) concentrations were significantly elevated (58%, *p* = 0.04) 24 h after the workout compared to the pre-exercise state. Similarly, the scale values of general, upper limbs, and lower limbs perceived recovery status were significantly lower 24 h post-exercise (39%, 12%, 47%, respectively, *p* = 0.013–0.046). Interestingly, after 48 h post-exercise, CK concentrations returned to baseline levels, and the scales values of perceived recovery status were significantly greater (*p* ≤ 0.05) compared with 24 h post-exercise. On the other hand, Martínez-Gómez et al. compared the effectiveness of three different recovery strategies: 1) low-intensity leg pedalling, 2) surface neuromuscular electrical stimulation (NMES), and 3) total (passive) rest after FFT. The authors concluded that, although there was a trend toward an improved perceived recovery with NMES compared with total rest (*p* = 0.061), low-intensity leg pedalling, NMES, and total rest promote a comparable recovery after a FFT session. These findings are of practical importance in real-world FFT since recovery status and strategies to improve recovery can help to optimize training monitoring while minimizing the potentially detrimental effects associated with the performance of repeated high-intensity efforts (Bishop et al., 2008; Balk and Englert, 2020).

## Adaptations of functional fitness training

FFT has been proposed to increase participants’ physical conditioning and performance with a broad range of fitness levels (Claudino et al., 2018; Feito et al., 2018; Schlegel, 2020; Sharp et al., 2022). Researchers have demonstrated that implementing FFT efficiently develops both strength and endurance adaptations in short-term and long-term programs (Feito et al., 2018; Schlegel, 2020; Sharp et al., 2022). Furthermore, the benefits can also be extended to psychosocial aspects since, elevated levels of sense of community, satisfaction, and motivation during FFT have commonly been reported in the literature (Claudino et al., 2018; Feito et al., 2018). Nonetheless, more research is needed to increase the knowledge on the resultant adaptations of implementing FFT in participants with different fitness levels. Additionally, it is essential to compare the effectiveness of FFT with other training programs.

In this Research Topic, two articles followed an intervention to elucidate the physiological adaptations of implementing FFT in men with different fitness levels (Sheykhlouvand et al.; Zuo et al., 2022). Besides, there was one correlational study that, although it was not a direct intervention, may be indicative of adaptations consequence of systematic FFT implementation (Mangine et al.). Finally, a randomized controlled trial of tangeretin supplementation on cortisol stress response induced by high-intensity resistance exercise was included (Liu et al., 2022).

Firstly, Sheykhlouvand et al. demonstrated that a new form of resistance-type HIIT (RHIIT) improved cardiac structure and hemodynamic, physiological adaptations, and performance of well-trained kayakers. In this study, twenty-four male kayakers were randomly assigned to one of three conditions (i.e., RHIIT, paddling-based HIIT [PHIIT], and a control group [CON]) for 8 weeks. Overall, RHIIT and PHIIT groups similarly improved cardiac structure, hemodynamic, other physiological parameters (e.g., maximal stroke volume, maximal oxygen uptake, maximal cardiac output, end-diastolic volume, ejection fraction, peak power output, and left ventricular end-systolic dimension, all *p* ≤ 0.05), and performance of well-trained kayakers (e.g., 500-m and 1000-m paddling performance, *p* ≤ 0.05). In addition, RHIIT group significantly improved maximum strength (*p* ≤ 0.05). Secondly, Zuo et al. demonstrated that functional resistance training (FRT) is as effective as traditional resistance training (TRT) for improving the upper and lower limb muscular endurance and performance in untrained young men. In this study, twenty-nine untrained men were randomly assigned to FRT or TRT for 6 weeks. The results of FRT and TRT groups showed equally significant increments in muscular endurance (*p* < 0.01) and performance (i.e., throwing, jumping, 30-m sprint and pull-ups performance, *p* < 0.01). Therefore, based upon the results of these studies, implementing RHIIT and FRT should be considered as efficient FFT alternatives to develop strength, muscular endurance, and cardiorespiratory adaptations in men with different fitness levels, as previously suggested (Feito et al., 2018; Schlegel, 2020).

Mangine et al. examined the relationships between body composition and FFT performance during the benchmark “FRAN”. In this cross-sectional study, fifty-seven men and thirty-eight women with different fitness levels completed a dual-energy X-ray absorptiometry assessment and were grouped by competition class (i.e., men, women, master’s men, master’s women) and percentile rank in the “FRAN” benchmark (i.e., ≤25th percentile, 25–75th percentiles, ≥ 75th percentile). The authors showed that “FRAN” performance varies by competition class and percentile rank in men and women. In men, greater body mass and bone mineral density were related to performance and muscle size, strength, and power (*p* ≤ 0.05). Meanwhile, body and skeletal mass were not related to “FRAN” performance in women. Across percentile ranks, the higher-ranking participants (≥75th percentile) had more non-bone lean mass and less body fat than all other participants, and those who had more lean mass performed better (*p* ≤ 0.05). Therefore, based upon the results of this study, it may be suggested that implementing FFT may increase non-bone lean mass - predominantly muscle mass - while reducing body fatness, especially in men.

Finally, Liu et al. (2022) conducted a randomized controlled trial to investigate the effects of 4 weeks tangeretin supplementation on the cortisol stress response induced by high-intensity resistance exercise (HIRE) in twenty-four male soccer players. Participants were randomly assigned to an experimental and a control group, all of them performing high-intensity bouts of resistance exercise to stimulate their cortisol stress responses. A dose of 200 mg/day of tangeretin was provided to the individuals of the experimental group while a placebo was ingested by those placed in the control group. The authors observed that 4 weeks of tangeretin supplementation can reduce serum cortisol and adreno-corticotropic hormone, and adaptation that could ameliorate the cortisol stress response in soccer players during high-intensity resistance exercise. In addition, tangeretin supplementation may also enhance antioxidant capacity, accelerate the elimination of inflammation, and shorten recovery time after high-intensity resistance exercise. Thus, 200 mg/day of tangeretin supplementation could mitigate the detrimental effects of cortisol stress response induced by FFT.

## Limitations and future perspectives

In this Research Topic, there have been several contributions for increasing the body of evidence FFT for health and performance including: 1) terminology, 2) acute effects of FFT, and 3) adaptations of FFT. However, there are several limitations in the studies published in this Research Topic. First, (Feito et al., 2018) studies still have no consensus on the terminology used to refer to FFT (e.g., CrossFit^®^, high-intensity functional training, high-intensity resistance training, functional resistance training, HIRE, HIIT using whole-body exercise), thus making difficult the consistency in the scientific literature and the consensus among researchers and practitioners. Second, (Claudino et al., 2018) most articles on this Research Topic have been conducted on healthy young, moderately trained men. Therefore, more studies with men and women of different ages with different biological conditions and fitness levels are warranted (Claudino et al., 2018; Feito et al., 2018; Tibana and Frade De Sousa, 2018). Unfortunately, due to the participants’ background in the studies covered by this Research Topic, caution must be taken when extrapolating the results to other populations. Third, (Bergeron et al., 2011) studies relating FFT performance to physiological and neuromuscular predictors are missed in this Research Topic. More research is needed to increase the knowledge of implementing FFT for health and performance, a training methodology increasingly gaining attention within the fitness community.
